# Identification of a Novel Lipid Metabolism-Associated Hepatic Gene Family Induced by Estrogen via ERα in Chicken (*Gallus gallus*)

**DOI:** 10.3389/fgene.2020.00271

**Published:** 2020-03-31

**Authors:** Hong Li, Yanmin Li, Liyu Yang, Dingding Zhang, Ziming Liu, Yanbin Wang, Ruili Han, Guoxi Li, Zhuanjian Li, Yadong Tian, Xiangtao Kang, Xiaojun Liu

**Affiliations:** College of Animal Science and Veterinary Medicine, Henan Agricultural University, Zhengzhou, China

**Keywords:** chicken, hepatocytes, lipid, expression regulation, estrogen, liver

## Abstract

Liver is the main organ of lipid metabolism in chicken, especially for laying hens. To explore the molecular mechanism of lipid metabolism in chicken, five novel genes discovered in chicken liver tissue were systematically studied. Bioinformatic analysis was used to analyze the gene characteristics. The expression patterns and regulatory molecular mechanism of the five genes were examined. Our results showed that all five novel genes contain a common NADP-binding site that belongs to the NADB-Rossmann superfamily, and the genes were designated *NADB-LER*1-5. Phylogenetic tree of the *NADB-LERs* gene family in different species suggested these five genes originated from the same ancestor. Tissue distributions showed that *NADB-LER1-4* genes were highly expressed in lipid metabolism organs, including liver, kidney and duodenum, and that the *NADB-LER5* gene was highly expressed in liver and kidney. The spatiotemporal expression indicated that the expression levels of *NADB-LER1-5* genes in liver tissue were significantly greater in sexually mature hens than that of immature pullets (*P*-value ≤ 0.05). The expression levels of *NADB-LER1-5* were significantly induced by 17β-estradiol in primary cultured chicken embryo hepatocytes (*P*-value ≤ 0.05), and 17β-estradiol regulated the expression of *NADB-LER1-5* mediated by ERα. Individual assays verified that under induction of 17β-estradiol, the five novel genes were significantly upregulated, with subsequent alteration in serum TG, TC, and VLDLs in 10-week-old pullets. This study proved *NADB-LERs* family mainly expressed in liver, kidney, and duodenum tissues. 17β-estradiol induces the expression of *NADB-LER1-5* genes predominantly mediated via ERα. They likely involved in lipid metabolism in the liver of chicken.

## Introduction

In chicken, liver is the most important and central site for lipid synthesis, responsible for more than 90% of total fatty acid *de novo* synthesis (O’Hea and Leveille, 1969). The hormonal control of lipid metabolism is a remarkable paradigm in laying hen. Studies have shown that estrogen could directly regulates the gene expression of chicken liver for yolk precursor synthesis (Bergink et al., 1974; Kirchgessner et al., 1987). Moreover, estrogen also is likely involved in regulating lipolytic activities and lipid transfer processes, which play important roles in the deposition of yolk into the fast-growing oocytes and in subsequent embryonic yolk utilization (Speake et al., 1998).

Especially during the egg-laying stage, under the influence of estrogen, many lipids including triacylglycerols, cholesteryl esters, and free fatty acids, are largely synthesized by the liver tissue. Then they are assembled into the egg-yolk precursors very low density lipoprotein (VLDL) and vitellogenin particles (Deeley et al., 1977), and transferred through blood circulation and deposited in the fast-growing oocyte (Brady et al., 1976; Wiskocil et al., 1980; Walzem et al., 1999; Nikolay et al., 2013). Therefore, lipid metabolism physiology is highly relevant to chicken embryonic growth development and the female reproductive system.

The lipid metabolism regulation process in poultry is highly similar to that in mammalian species, while recent work found that the functions of some genes and their products in poultry are different from mammals (Wiskocil et al., 1980; Kirchgessner et al., 1987; Hermier et al., 1996; Mason, 1999). Moreover, a recent study had demonstrated that some of the genes involved in lipid metabolism (e.g., resistin, *TNF*α, and *SERPINE1*) in poultry species, compared with mammals, may have been lost during the evolutionary process (Daković et al., 2014). Therefore, the hepatic lipid metabolism regulation mechanism of chicken needs to be elucidated.

Compared to other gene expression profile technology, RNA-seq is a superior method in analyzing gene expression at different development periods or in different tissues in a single assay, and it can help in the discovery of novel genes and splice variants (Trapnell et al., 2012). Previous study on liver tissue of chickens at the pre- and peak-laying stages by RNA-seq revealed the differentially expressed genes mainly involved in regulating lipid metabolism-related processes, and may do the same of the novel discovered protein-coding genes (Li et al., 2015).

A novel uncharacterized protein-coding gene family with five members was differentially expressed in the liver of hens between these two physiological stages (Li et al., 2015). According to bioinformatic analysis, the five members contain the same highly conserved NADP(H)-binding motif and the same substrate specificity site, which belong to NADB-Rossmann superfamily (Hanukoglu, 2015). Our subsequent study showed that the five NADB members with relatively high abundant expressed in chicken liver tissue were regulated by 17β-estradiol and were named *NADB-LER1*, *NADB-LER2*, *NADB-LER3*, *NADB-LER4*, and *NADB-LER5*. In addition, a β-ketoacyl acyl carrier protein reductase (FabG) component and an adh_short domain were also discovered among these novel genes. FabG, an NADPH-dependent β-ketoacyl-ACP reductase (Hoang et al., 2002), is an essential and universally expressed component of type II fatty acid biosynthesis (Zhang et al., 2003). To understand the biological functions of these genes, the cDNA sequences of the five mRNAs were cloned, and the phylogenetic relationship among different species was analyzed. Their tissue expression pattern and expression regulation mechanism were systematically investigated at *in vivo* and *in vitro* levels, respectively.

This report provides the first identification and expression profiling of *NADB-LERs* in chicken. The findings suggested that these newly identified proteins function in processes controlling lipid metabolism in the liver of chicken.

## Materials and Methods

### Ethics Statement

All the animal experiments were performed according to the Institutional Animal Care and Use Committee guidelines following protocols approved by the Henan Agricultural University (Permit Number: 11-0085).

### Animals and Treatments

All the birds used in this study were provided by the Center of Poultry Germplasm Resource of Henan Agricultural University. They were raised in the same environmental conditions with *ad libitum* access to food and water.

The Lushi blue-shelled-egg chickens were used as the study animals. Hens at the pre-laying (20 week) stage without start laying and peak-laying (30 week) stage with an egg laying rate of over 70% were used (Li et al., 2015). Six hens at each stage were slaughtered and 11 tissues including the heart, liver, spleen, lung, kidney, duodenum, pectoral, abdominal fat, glandular stomach, pancreas and ovary tissues were collected, snap-frozen in liquid nitrogen and stored at −80°C until use. In addition, six female birds from the flock hatched in the same batch were slaughtered at each of the five different time points (1-day-old, 1-, 10-, 15-, and 35-week-old), and 11 tissues were collected and stored as mentioned above.

Sixty-four healthy female Lushi blue-shelled-egg chickens at the age of 10 weeks with similar body weights were divided into 4 groups with 16 birds in each group. The birds in the control group received an intramuscular injection of (specify volume) olive oil. The birds in the three experimental groups received the intramuscular injection of 0.5, 1.0, and 2.0 mg/kg body weight of 17β-estradiol (Sigma, St. Louis, MO, United States) (dissolved in olive oil), respectively. After injection for 12 h, eight birds from each group were randomly selected, and 3–5 mL blood sample of each bird was collected from wing vein. Then, the chickens were humanely slaughtered. Subsequently, the livers and kidneys were collected and stored as described above. The remaining eight birds in each group were continuously fed, and humanely killed until 24 h after injection. The blood samples, livers and kidneys were collected and stored as mentioned above.

### Blood Physiological and Biochemical Index Measurement

The serum samples were collected from the fresh whole blood at room temperature for 1 h, then centrifuged at 2000 *g* for 15 min and stored at −70°C until further analysis. Blood physiological indexes of serum samples, including the low-density lipoprotein cholesterol (LDL-c) (Jiancheng, Corp., Nanjing, China) and high-density lipoprotein cholesterol (HDL-c) (Jiancheng), total cholesterol (TC) (Jiancheng), and triglyceride (TG) (Jiancheng), were determined using a blood analyzer (Hitachi 7100), while the very low-density lipoprotein cholesterol (VLDL-c) could instead be determined by the cholesterol contained in the VLDL and was indirectly determined by the Friedewald formula:VLDL-c = TC-HDL-c-LDL-c (Friedewald et al., 1972; Delong et al., 1986).

### Cell Culture and Stimulation

The specific-pathogen-free (SPF) fertilized eggs purchased from Beijing Meiliyaweitong Experimental Animal Technology, Co., Ltd. (Beijing, China) were incubated in a sterile environment under suitable conditions (Ma et al., 2017). When the fertilized eggs were incubated for 18-day-old subjects, the embryonic primary hepatocytes were isolated from the liver tissue of the embryos according to a modified protocol based on the method put forward by Fischer and Marks (1976). In brief, the prepared hepatocytes were seeded in six-well plates and cultured in William’s E medium (Sigma, St. Louis, MO, United States) with 10% fetal bovine serum (FBS), 100 U/mL penicillin, and 100 mg/mL streptomycin. Hepatocytes were incubated at 37°C under a water-saturated atmosphere containing 95% air humidity and 5% CO_2_ overnight.

When the hepatocytes were grown to 70–80% confluence and serum-starved for 12 h, cells were exposed to 0, 25, 50, and 100 nM 17β-estradiol dissolved in olive oil, with six replicates for each treatment concentration. After stimulation for 12 h, the cells were collected and stored at −80°C for further experiments. This assay was repeated independently three times.

Different estrogen receptor antagonists, including methyl-piperidino-pyrazole (MPP), an estrogen receptor alpha (ERα)-selective antagonist, and ICI 182,780 and tamoxifen (Sigma-Aldrich), which are antagonists of both ERα and ERβ and may also act as the agonists of GPR30 on certain genes in some species (Guo et al., 2006; Pang et al., 2008), were dissolved in dimethyl sulfoxide (DMSO). When the cells were prepared in six-well plates for experiments, 17β-estradiol and 17β-estradiol combined with 1 μM MPP, 1 μM ICI 182,780, or 1 μM tamoxifen were used to treat the hepatocytes. Each treatment group had six replicates. The control group suffered from olive oil and DMSO at a final concentration of 0.1% each. After treatment for 24 h, cells were collected and stored at −80°C until use. The experiment was repeated independently three times.

### Gene Cloning and Sequencing

Total RNA was extracted from cells and tissues using TRIzol reagent according to the manufacturer’s protocol (Invitrogen, Carlsbad, CA, United States). Transcription of 1 μg of RNA into cDNA was performed using the Thermo ScientificTM Revert Aid First Strand cDNA Synthesis kit (Thermo Scientific) according to the manufacturer’s instructions. The cDNA samples were stored at −20°C for further analysis.

To understand the structures and potential biological functions of novel genes available from previous RNA-seq data (Li et al., 2015), bioinformatic analysis was performed to analyze the protein conserved domains of 91 up-regulated expressed novel genes. Five of these differentially expressed genes (ENSGALG00000001824, ENSGALG00000023936, ENSGALG00000021451, ENSGALG00000021450, and ENSGALG00000001791) after named *NADB-LER1-5* were further studied.

According to the predicted chicken *NADB-LER1-5* sequences published in the Ensemble databank, several primer pairs were designed to produce overlapping fragments, and finally, the eight pairs of specific PCR primers in Table 1 were used for cloning the complete coding sequences (CDs) of the five mRNAs. Primers were designed using the Primer 5.0 software. PCRs were carried out in a total reaction volume of 25 μL, and the reaction component consisted of 1.0 μL of cDNA sample, 12.5 μL of 2 × pfu MasterMix (Tiangen Biotech, Co., Ltd., Beijing, China), 1.0 μL each of the forward and reverse primers (10 μM), and 9.5 μL of RNase-free water. The amplification reaction procedure was the following: an initial denaturation at 95°C for 5 min; followed by 35 cycles, with each cycle consisting of 30 s at 95°C, 30 s at 60°C and 30 s at 72°C; followed by a final elongation step at 72°C for 10 min. PCR products detected with the right fragment size were sequenced by Sangon Biotech, Co., Ltd. (Shanghai, China). Each sequence was sequenced on both strands.

**TABLE 1:** Primers used for gene clone.

Primer name	Primer sequence (5′–3′)	Product size (bp)
*NADB-LER1-1*	F: GAGTGGGTCTTTGCAACCTG	635
	R: AGAGGTTGGAGAGCACCTTC	
*NADB-LER1-2*	F: TTTGTTTCCGCTCCGTTCT	369
	R: GCGATGGTGTTGGTGGTG	
*NADB-LER2-1*	F: CTGCCGCTCCGTGCTCAT	753
	R: TTTCCCCTTGCCAGTCCA	
*NADB-LER2-2*	F: GCCGCGCTGCCGCTCCGT	286
	R: ATTGATGCCAGCGTTGTT	
*NADB-LER3*	F: ATTCGGGCACTGAGCAAA	847
	R: GCACAACTCCGTGTAGCA	
*NADB-LER4*	F: CTGAGCAAACAACACGAGCT	1028
	R: AGTGAGACGCTGTTGGAAGA	
*NADB-LER5-1*	F: CAAGGCAGCCATCATCAA	346
	R: CACTGTGTGGTCCCTCAAAT	
*NADB-LER5-2*	F: ATCCAAACACCCAAACCTG	635
	R: CACTGTGTGGTCCCTCAAAT	

### Bioinformatic Analysis

To predict the protein structure and potential function of the five members, online databanks and software were used. The NCBI^1^ was used to predict the conserved domains. PSORT^2^ was used to analyze the subcellular localization. SignalP^3^ was used to predict the signal peptides. TMHMM^4^ was used to predict the membrane-spanning domains. ProtFun^5^ was used to predict the protein functions.

The complete CD sequences of the five members were obtained and used for phylogenetic analysis. The *NADB-LER 1-5* nucleotide sequence alignments were performed using Clustal X2 (Thompson et al., 1994). Phylogenetic analyses of the five members among different species was constructed using the neighbor-joining method. Amino acid alignments were generated using Clustal V in the Molecular Evolutionary Genetics Analysis version 6.0 (MEGA 6) software (Tamura et al., 2013). The motif of the five genes were also predicted using online software MEME5.0.5.^6^ The evolutionary model was set to the Tamura 3-parameter model with a proportion of invariant sites and discrete gamma model implemented in PAUP^∗^ software (version 4.0b10) (Swofford, 2002). Evaluation of statistical confidence in nodes was based on 2000 bootstrap replicates. All branches with a bootstrap value equal to or greater than 50% are shown, while others were collapsed.

### Quantitative Real-Time PCR (qPCR)

The oligonucleotide primers used for qPCR are shown in Table 2. All the primers were designed using the principle of span exons to avoid genomic DNA contamination. The qPCR was carried out on the LightCycler96 real-time PCR system (Roche Applied Science, Penzberg, Germany). Each reaction was performed in a final reaction volume of 10 μL containing 1 μL of cDNA, 5 μL of 2 × SYBR Green PCR Master Mix (Takara), 0.5 μL each of forward and reverse primer (10 μM) and 3 μL of RNase-free water. The qPCR amplification procedure was as follows: preincubation at 95°C for 5 min, and followed by 40 cycles of 30 s at 95°C, 30 s at 60°C and 30 s at 72°C. β-Actin mRNA was used as the housekeeping gene. *ApoVLDL II*, with the canonical estrogen response element (ERE), is an estrogen-dependent marker gene, is liver specific (Ryan et al., 1994) and was used as the positive control. All samples were analyzed in triplicate, and the average value of the triplicates was used for quantification.

**TABLE 2:** Primers used for qPCR.

Gene name	Primer sequence (5′–3′)	Product length (bp)
*NADB-LER1*	F: GGCCATCGTCAACATTTCCA	142
	R: CCATGTCTGTTTGCACCCAT	
*NADB-LER2*	F: TGCCGCTACCCCGATAAG	151
	R: ACATTGATGCCAGCGTTGTT	
*NADB-LER3*	F: CATTGCCAAACCCACCTGAG	155
	R: CAGCGTTGTTGATGAGGAGG	
*NADB-LER4*	F: TCAACAACGCTGGAATTGCA	149
	R: GGCAGTGCTGGAAATGTTGA	
*NADB-LER5*	F: GATAAACAACGCCGGCATCT	166
	R: CTTCTTCAAGAGGGGCAGGA	
β*-Actin*	F: CAGCCAGCCATGGATGATGA	118
	R: ACCAACCATCACACCCTGAT	
*ApoVLDLII*	F: CAATGAAACGGCTAGACTCA	108
	R: AACACCGACTTTTCTTCCAA	
*ApoB*	F: TTATGCAACGCTTCAGGTTC	136
	R: TCCATTGAGACGAGCATT	

### Statistical Analysis

Gene expression levels were normalized to β*-actin* mRNAs and analyzed using the 2^–^^ΔΔ*ct*^ method (Schmittgen and Livak, 2008). The One-Way Analysis of Variance followed by the Student’s *t*-test of the GraphPad Prism, version 5 (Graph Pad Software, Inc., San Diego, CA, United States) was used to determine the statistical significance. Due to the cDNA sample of each tissue used for expression distribution is a mixture at an equal volume of six individuals, therefore the data without significant analysis. Besides, all the rest values are presented as the mean ± SE, and differences with *P-*value ≤ 0.05 were considered statistically significant.

## Results

### Identification and Sequencing of Novel Genes in Chicken

**FIGURE 1:**
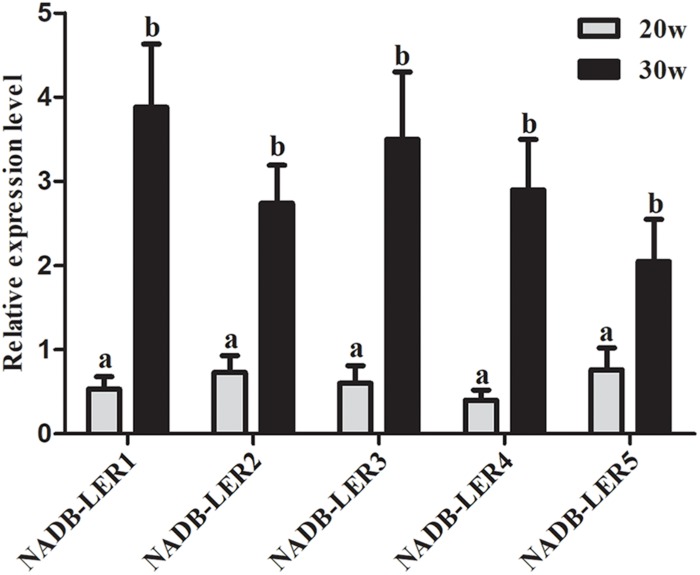
The relative expression levels of *NADB-LERs* mRNAs in the livers of chickens at the age of 20- and 30-week-old. a, b different letters indicate a significant difference between groups (*P-*value ≤ 0.05) (*n* = 6).

To understand the structures and potential biological functions of NADB-LER1-5, bioinformatic analysis was performed. The five genes had the same NADP-binding motif and a substrate specific binding site shared by the NADB-Rossmann superfamily (Zhong et al., 2014). The sequence similarity of NADP-binding motifs ranged from 49.21 to 88.54%. In addition, a specific β-ketoacyl acyl carrier protein reductase (FabG) component, an Adh_short domain and a DItE domain were also discovered among the five novel genes (Supplementary Figure S1). According to the qPCR analysis, the five genes are significantly up-regulated in the liver tissue of 30 week hens relative to 20 week hens, which is in according with the RNA-seq data (Figure 1).

**FIGURE 2:**
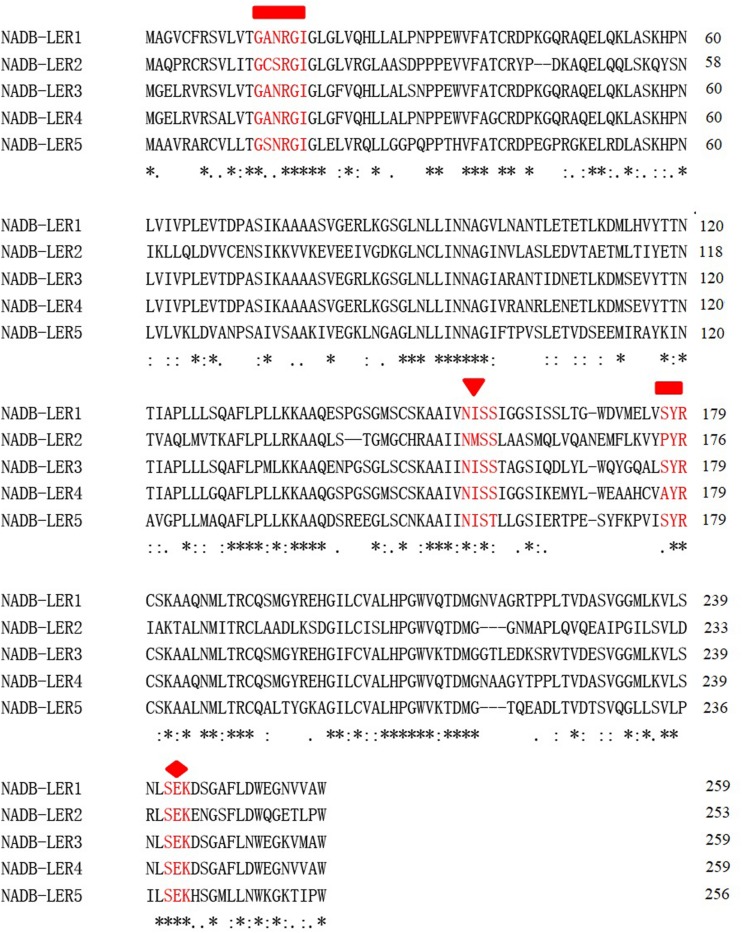
Amino acid sequence alignment of chicken NADB-LERs by Clustal X2. The relatively conserved N-myristoylation, N-glycosylation, and protein kinase C phosphorylation sites are marked by rectangles, triangles and rhombuses, respectively. Amino acids identical in all five proteins are marked with an asterisk (^∗^), conservative substitutions with a colon (:), and semiconservative substitutions with a period (.).

To identify novel chicken NADB-LERs, the draft chicken genomic database was queried using the predicted sequences published in the Ensemble databank. The PCR was performed to amplify the identified sequences using chicken liver tissue cDNA as template. The resulting DNA fragments were cloned and sequenced. By this approach, the full-length cDNA cloned from the chicken genes corresponding to *NADB-LER1, NADB-LER2, NADB-LER3, NADB-LER4*, and *NADB-LER5* were obtained, and they are the same as the predicted sequences published in the Ensemble databank. They were found on chicken chromosomes 11 (*NADB-LER1, -3, -4*, and *-5*) and 21 (*NADB-LER2*). The subcellular localization analysis showed that *NADB-LER1* is located extracellularly and the other four members are located on the cell membrane. In addition, none of them has a signal peptide or a membrane-spanning domain (Table 3). The amino acid alignment of the CDSs of the five chicken NADB-LERs is shown in Figure 2. The sequence identity is relatively high, and the N-myristoylation, N-glycosylation, and protein kinase C phosphorylation sites are well-conserved among the members.

**TABLE 3:** The prediction of subcellular locations, signal peptide, and membrane spanning domain.

Novel gene	Gene id	Location	Amino acids length	Subcellular locations	Signal peptide	Membrane spanning domain
NADB-LER1	ENSGALG00000001824	11:1155406-1157865	259 aa	Extracellular	No	No
NADB-LER2	ENSGALG00000023936	21:2227460-2235534	253 aa	Cytomembrane	No	No
NADB-LER3	ENSGALG00000021451	11:1039249-1041839	259 aa	Cytomembrane	No	No
NADB-LER4	ENSGALG00000021450	11:1044294-1057861	259 aa	Cytomembrane	No	No
NADB-LER5	ENSGALG00000001791	11:1064190-1067062	256 aa	Cytomembrane	No	No

### Phylogenetic Analysis

**FIGURE 3:**
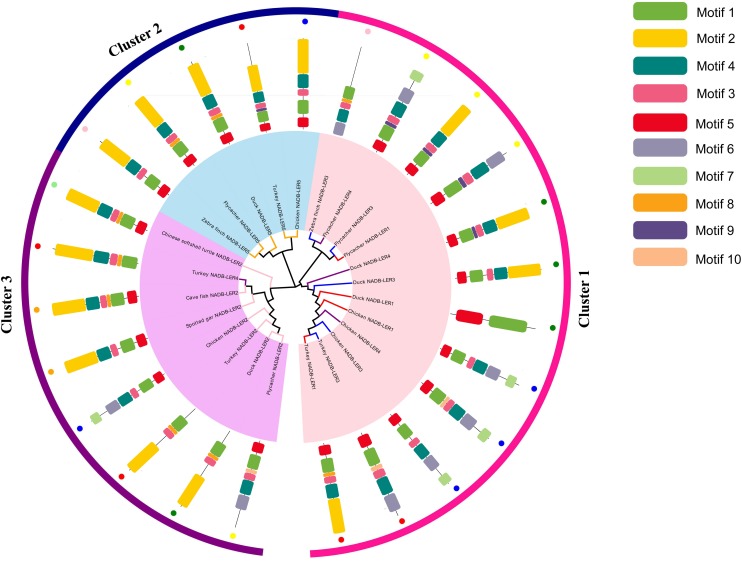
Consensus phylogenetic tree of *NADB-LER*s among different species. Different small color dots mean different species; the different color branches mean different members, except black.

To investigate the molecular function evolution and the relationships among the homologs, the NADB-LER amino acid sequences from chicken were aligned using phylogenetic methods. A BLAST search found that the novel genes only were presented in avian and other lower vertebrates. Multiple alignments of *NADB-LER* amino acid sequences from different species, including chicken and seven other representative species from avian, batrachians, reptiles and fish, were obtained by Clustal X2. The Ensemble IDs for each gene from this range of species are listed in Supplementary Table S1. The evolution analysis of these genes from different species showed that the phylogeny tree was divided into three clusters by *NADB-LER2*, *NADB-LER5* and the other three members, and they belong to paralogs of the other three genes, while *NADB-LER1*, *NADB-LER 3*, and *NADB-LER 4* are relative low homologous among different species, and divided into two small branches. In each cluster, they are almost own the similarity motifs (Figure 3).

### Expression Profiles

**FIGURE 4:**
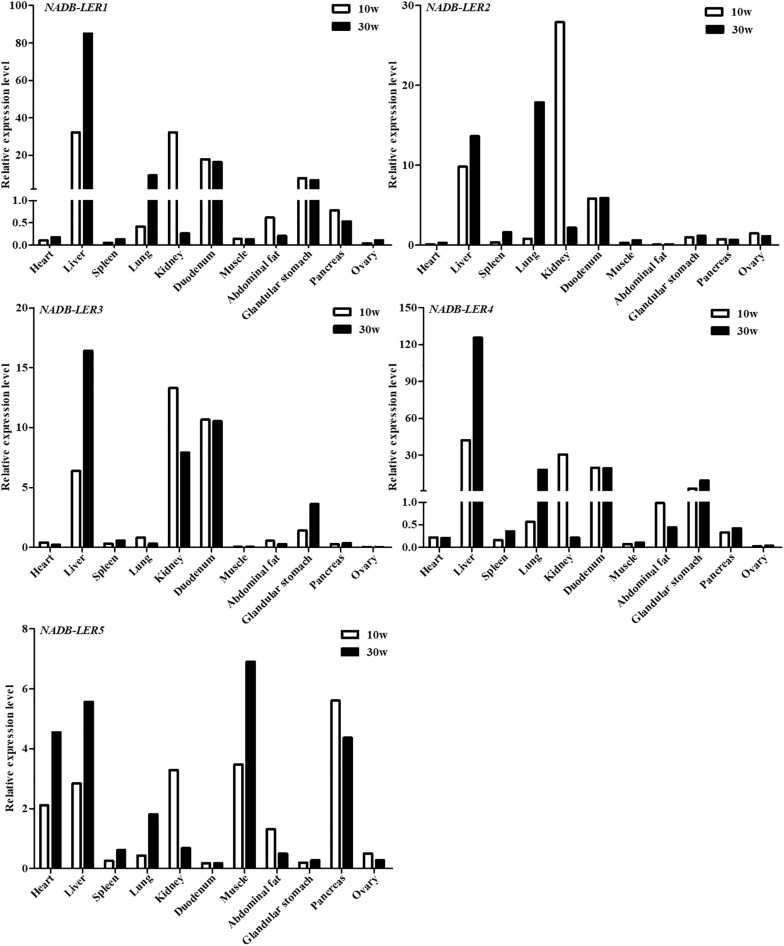
The relative expression levels of *NADB-LER*s in different tissues of 10- and 30-week-old chickens. *n* = 1, a cDNA pool of six individuals for each tissue sample was used as temple.

To analyze the tissue expression profiles of the above-described *NADB-LER*s, the mRNA relative expression levels in 11 chicken tissues by qPCR were tested (Figure 4). The cDNA pool for each tissue mixed with six individuals was used as the temple. The results showed the five genes are expressed predominantly in liver tissue and expressed to a greater degree in 30-week-old hens than in 10-week-old hens. Relatively high expression levels of these genes were also measured in kidney at 10 weeks. The expression patterns of *NADB-LER1* and *NADB-LER4* resembled each other in different tissues. Duodenum expressed relatively high levels of *NADB-LER1, -LER2, -LER3*, and *-LER4* and almost presented the same levels in 10- and 30-week-old hens. Lung expressed relatively high levels of *NADB-LER1, -LER2, -LER4*, and *LER5*, while the relative level is higher in 30-week-old than in 10-week-old hens. In contrast, heart, muscle and pancreas expressed relatively high levels of *NADB-LER5* in 30-week-old hens.

**FIGURE 5:**
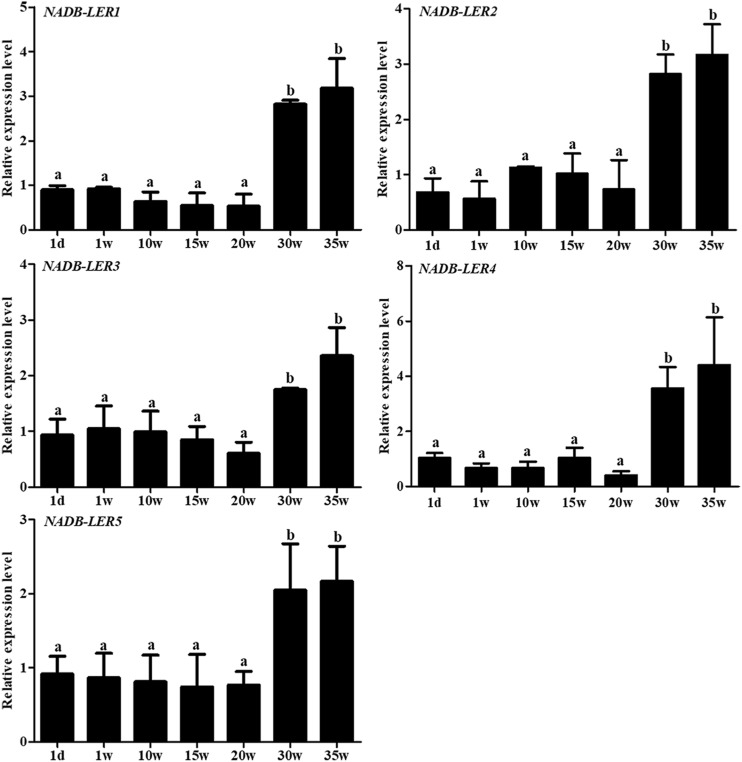
The relative expression levels of *NADB-LERs* genes in liver of chicken at different development stages. Different letters indicate a significant difference between groups (*P-*value ≤ 0.05). For each treatment, the data are expressed means ± SE (*n* = 6).

To gain insight into the spatiotemporal expression of *NADB-LER*s in the liver tissue of egg-laying chickens, their relative mRNA expression levels at seven different growth development stages were analyzed (Figure 5). Unexpectedly, the expression patterns of *NADB-LER*s resembled each other. In greater detail, the mRNA expression of the *NADB-LER*s at the peak-laying stages (30 and 35 week) were significantly higher than those of pre-laying stages (*P*-value ≤ 0.05), and there was no significant difference between the expression levels in 30- and 35-week-old hens (*P-*value > 0.05). Significant differences in *NADB-LER* expression levels were also not found among 1 day, 1-, 10-, 15- and 20-week-old hens (*P*-value > 0.05).

### Regulation of Novel Chicken Genes by Estradiol *in vitro*

**FIGURE 6:**
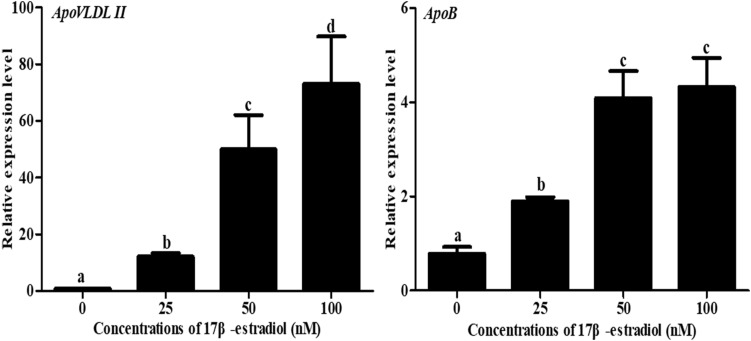
Effects of different doses of 17β-estradiol on the expression of *ApoB* and *ApoVLDL II* in primary hepatocytes after treatment for 12 h. Different letters indicate a significant difference (*P*-value ≤ 0.05). For each treatment, the data are expressed means ± SE (*n* = 6).

**FIGURE 7:**
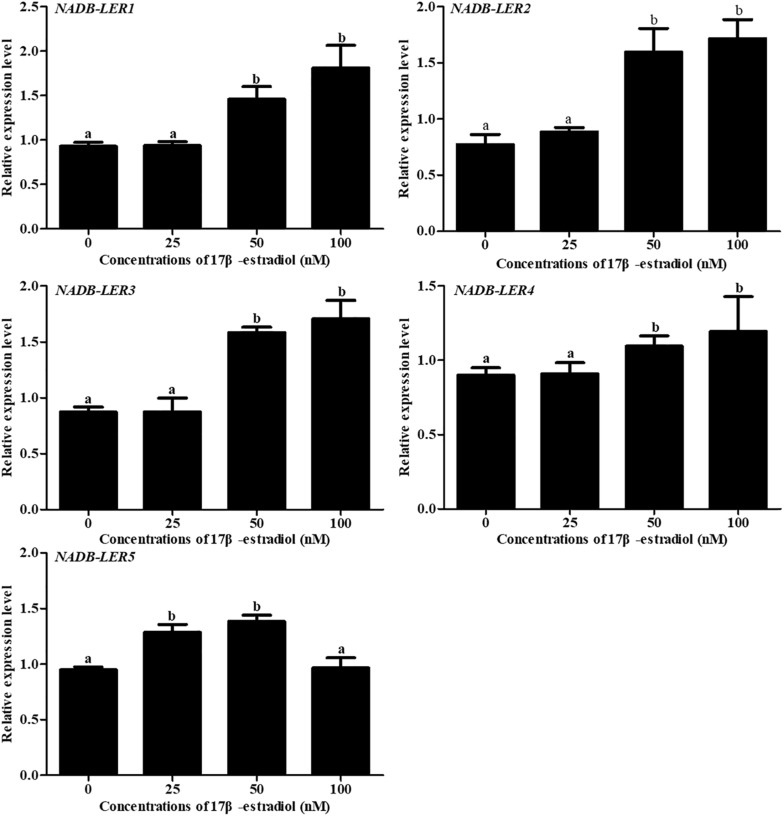
The effects of different doses of 17β-estradiol on the expression of *NADB-LERs* in primary hepatocytes after treatment for 12 h. Different letters indicate a significant difference (*P*-value ≤ 0.05). For each treatment, the data are expressed means ± SE (*n* = 6).

To understand the effect of 17β-estradiol on the expression of *NADB-LER*s *in vitro*, all the mRNA expression levels of the family members were analyzed in chicken embryonic primary hepatocytes stimulated with 17β-estradiol. The results showed that compared with their levels in the control group, both *ApoVLDL II* and *ApoB* genes were significantly upregulated by different concentrations of estradiol, and they presented dose-dependent expression (*P-*value ≤ 0.05) in chicken hepatocytes (Figure 6). Compared with their levels in the control, the mRNA expression levels of *NADB-LER1-4* in chicken hepatocytes were significantly increased under induction by estradiol at 50 and 100 nM concentrations (*P-*value ≤ 0.05), while there was no significant difference between the two treatment groups (*P-*value > 0.05). Different from the levels of the other members, the *NADB-LER5* mRNA expression level was significantly upregulated at 25 and 50 nM concentrations (*P-*value ≤ 0.05) but returned to the control level when cells were treated with 100 nM 17β-estradiol (*P*-value > 0.05) (Figure 7). In addition, 50 nM was the optimal concentration that induced the expression of *NADB-LER*s.

**FIGURE 8:**
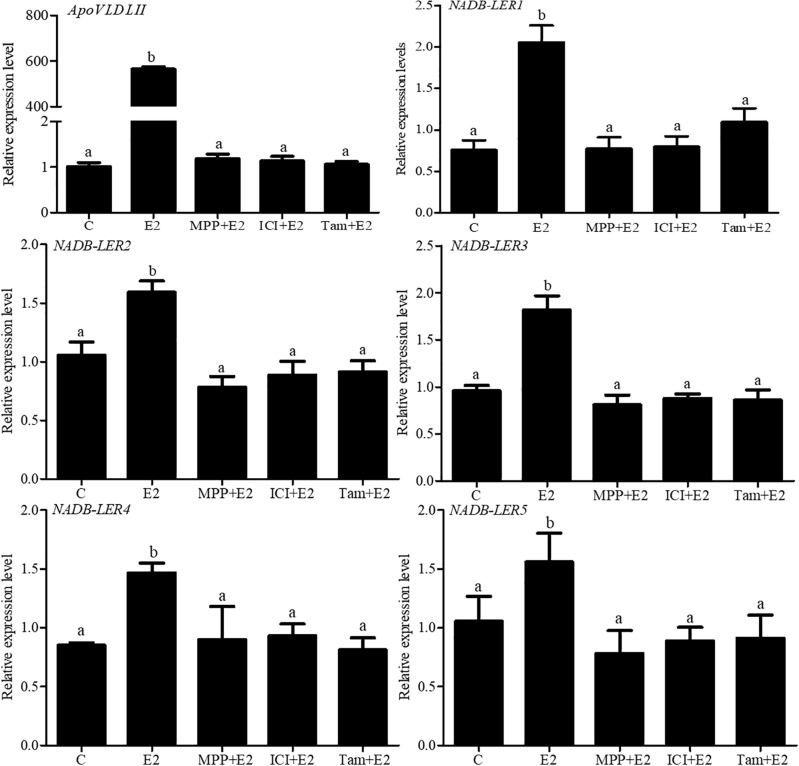
Effects of ER antagonists on the expression of *NADB-LER1-5* in chicken embryo hepatocytes. C: the control group; E2:17β-estradiol (50 nM); ICI: ICI182,780 (1 μM), TAX: tamoxifen (1 μM), and MPP: (1,3-*bis* (4-hydroxyphenyl) -4-methyl-5-[4-(2-piperidinylethoxy) phenol]-1H-pyrazoledihydrochloride) (1 μM). Different letters indicate significant difference among the different treatments (*P*-value ≤ 0.05). For each treatment, the data are expressed means ± SE (*n* = 6).

To further analyze the estrogen receptor (ER) subtype that mediates the expression of *NADB-LER* genes, chicken embryo primary hepatocytes were treated with 17β-estradiol alone or combined with different ER-subtype antagonists (Figure 8). As a positive control, compared with the 50 nM 17β-estradiol treated group, the expression of *ApoVLDL II* was significantly down-regulated when the hepatocytes were treated with 50 nM 17 β-estradiol combined with 1 μM MPP (*P*-value ≤ 0.05), and there was no significant difference among the groups treated with 50 nM 17 β-estradiol combined with either 1 μM MPP, or 1 μM tamoxifen or 1 μM ICI 182,780 (*P-*value > 0.05). Similarly, the expression levels of *NADB-LER*s were significantly decreased (*P-*value ≤ 0.05) when the hepatocytes were treated with 50 nM 17 β-estradiol combined with 1 μM MPP or 50 nM 17 β-estradiol combined with 1 μM tamoxifen and 1 μM ICI 182,780 (*P-*value ≤ 0.05). The expression levels of the genes returned to the control level in the three ER subtype antagonist-treated groups (*P*-value > 0.05). The results revealed that estrogen effects on the expression of chicken hepatic *NADB-LER*s were mediated by ERα.

### Estradiol Regulation *in vivo*

**FIGURE 9:**
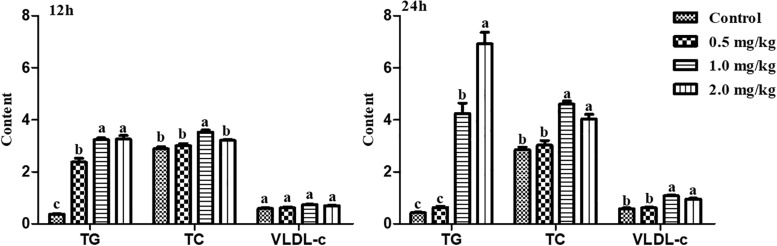
The differences of TG, TC, and VLDL-c contents in chicken serum treated with different concentrations of 17β-estradiol for 12 and 24 h, respectively. Different letters indicate a significant difference (*P-*value ≤ 0.05). For each treatment, the data are expressed as the means ± SE (*n* = 8).

**FIGURE 10:**
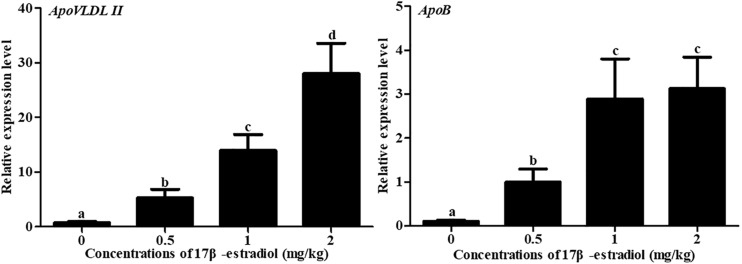
Effects of different doses of 17β-estradiol on the expression of *ApoB* and *ApoVLDL II* in the livers of 10-week-old pullets after treatment for 24 h. Different letters indicate a significant difference (*P*-value ≤ 0.05). For each treatment, the data are expressed as the means ± SE (*n* = 8).

To further validate whether estrogen influenced the expression of chicken *NADB-LER*s in individuals, female pullets at the age of 10 weeks were treated with different doses of 17β-estradiol for 12 h and 24 h. The blood biochemical index of the chickens was measured, and the results showed estrogen could significantly increase the concentrations of TG and TC when applied for 12 h (*P-*value ≤ 0.05), while the concentration of VLDLs was not changed (*P*-value > 0.05). Twenty-four hours of 17β-estradiol treatment at concentrations of 1 and 2 mg/kg dramatically increased the concentrations of TG, TC, and VLDLs (*P*-value ≤ 0.05) (Figure 9). Therefore, the optimal response time to 17β-estradiol for chicken was 24 h. In addition, both the expression of *ApoVLDL II* and *ApoB* genes were significantly increased in a dosage-dependent manner after treatment with 17β-estradiol for 24 h (*P*-value ≤ 0.05) (Figure 10).

**FIGURE 11:**
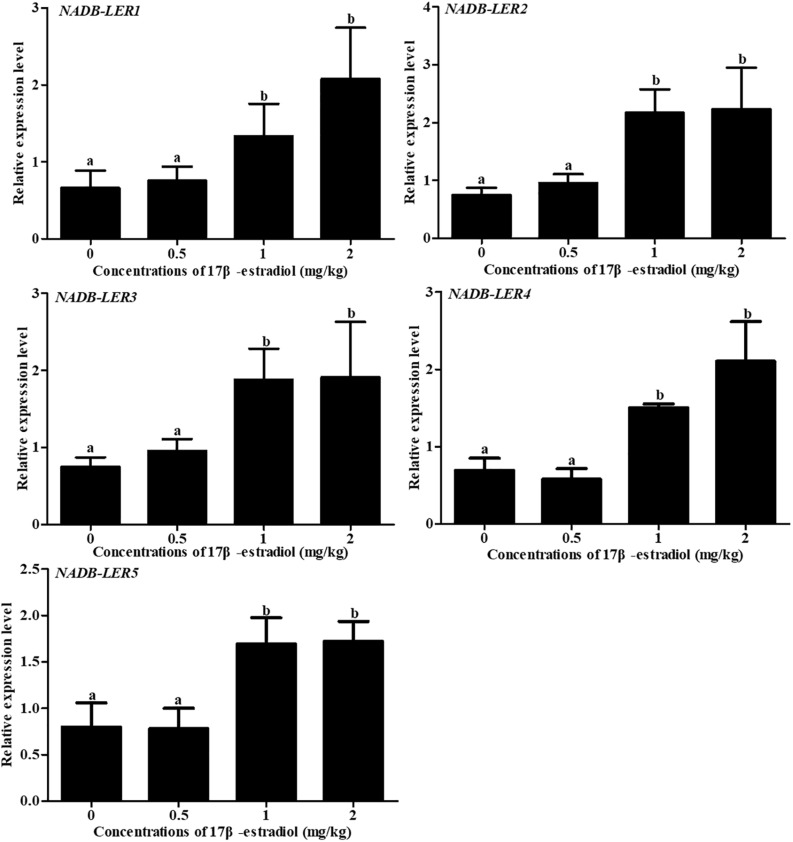
Effects of different doses of 17β-estradiol on the expression of *NADB-LERs* in the livers of 10-week-old pullets after treatment for 24 h. Different letters in each figure indicate a significant difference (*P-*value ≤ 0.05). For each treatment, the data are expressed as the means ± SE (*n* = 8).

To analyze the effect of 17β-estradiol on *NADB-LER*s in chicken liver tissue, the mRNA expression levels of *NADB-LER*s were analyzed in the liver of chicken treated with 17β-estradiol for 24 h. Compared to the levels in the control group, the relative expression levels of the five genes were significantly up-regulated at treatment concentrations of 1 and 2 mg/kg (*P-*value ≤ 0.05), while no significant difference was found between the two groups (*P*-value > 0.05); the expression levels of the five genes at a treatment concentration of 0.5 mg/kg were not changed from their levels in the controls (*P*-value > 0.05) (Figure 11).

**FIGURE 12:**
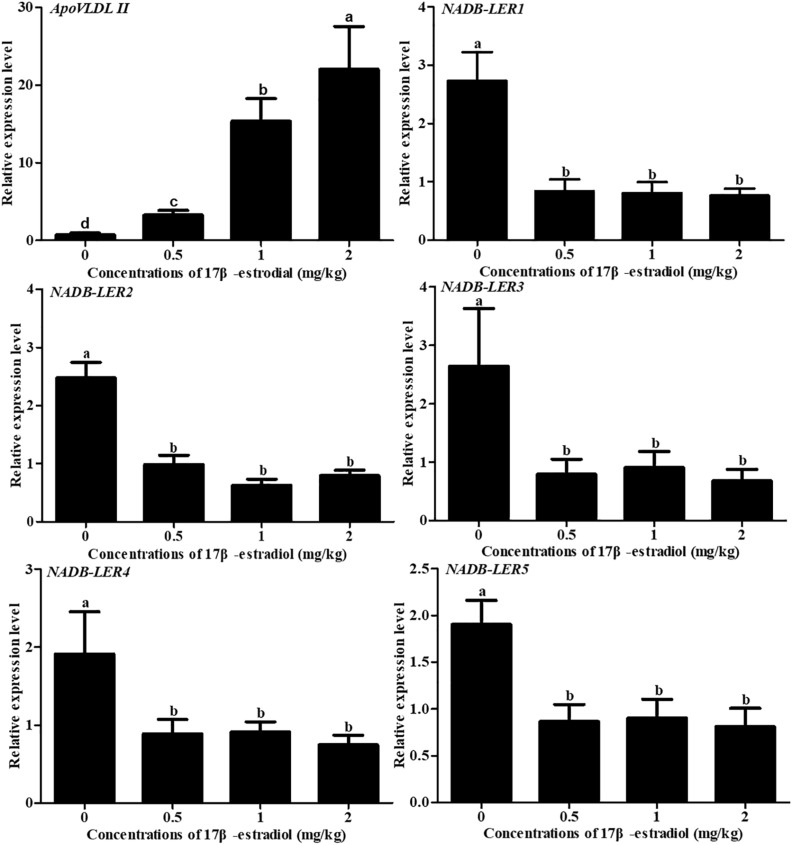
Effects of different doses of 17β-estradiol on the expression of *ApoVLDL II* and *NADB-LERs* in the kidney of 10-week-old pullets after being treated for 24 h. Different letters in each figure indicate a significant difference (*P-*value ≤ 0.05). For each treatment, the data are expressed as the means ± SE (*n* = 8).

To understand the effect of estrogen on *NADB-LER*s in chicken kidney, the mRNA expression levels of *NADB-LER*s were analyzed in the kidney tissue of chicken treated with estrogen for 24 h. Meanwhile, the expression of the *ApoVLDL II* gene was also analyzed. Compared to its level in the control group, *ApoVLDL II* was significantly up-regulated by different concentrations of 17β-estradiol and presented dose-dependent expression (*P*-value ≤ 0.05). In contrast, the relative expression levels of the *NADB-LER*s were significantly down-regulated from their control levels at different treatment concentrations (*P-*value ≤ 0.05), while no significant difference was found among the three treatment groups (*P*-value ≤ 0.05) (Figure 12).

## Discussion

In this study, five differentially expressed novel genes called *NADB-LERs* were found basing on a previous RNA-seq experiment (Li et al., 2015). Our study first systemically explored the characteristics of the NADB_Rossmann superfamily in chicken. The *NADB-LER* members were identified to contain the same conserved NADB-Rossmann structural domain and special component FabG using bioinformatics. Phylogenetic tree analysis suggested that the *NADB-LER1-5* genes originated from the same ancestor. Therefore, the *NADB-LER*s may have been generated through gene duplication, and different functions were produced in the process of evolution.

FabG, an essential and universally expressed component of type II fatty acid biosynthesis, appeared in *NADB-LER*s. FabG enzymes are members of the short-chain alcohol dehydrogenase/reductase (SDR) family (Nanson and Forwood, 2015). In addition, SDR enzymes play critical roles in lipid, amino acid, carbohydrate, cofactor, hormone, and xenobiotic metabolism and in redox sensor mechanisms (Mayoral et al., 2013). Five *NADB-LER* members were significantly up-regulated in the liver of peak-laying hens compared to pre-laying female chickens (Li et al., 2015). The most significant physiological difference between 20- and 30-week-old layer hens is whether they lay eggs or not; during the egg-laying period, to supply sufficient nutrients for embryo growth and development, neutral fatty acids, mainly triacylglycerols, are synthesized in the liver of hen under promotion by estrogen (Bergink et al., 1974). It was suggested that the five novel genes are related to lipid metabolism.

The five genes were extensively expressed in various tissues, while the high levels of *NADB-LER1, -2, -3*, and *-4* in the liver, kidney and duodenum of chicken may indicate tissue-specific role(s) of these genes. Liver, kidney and duodenum, as the main lipid metabolism organs in chicken, undertake different metabolism roles. VLDLy, intermediate-density lipoprotein (IDL) and low-density lipoprotein (LDL) are mainly synthesized and secreted in liver, chyle particles (CM) are mainly synthesized in small intestine, and VLDL and high-density lipoprotein (HDL) are mainly synthesized in and secreted from kidney (Bensadoun and Rothfeld, 1972; Tarugi et al., 1998). Moreover, in chicken, most of the yolk protein, including apolipoproteins and vitellogenins, are synthesized by the liver tissue (Deeley et al., 1977). The relative mRNA levels of *NADB-LER1, -2, -3, -4*, and *-5* were clearly more highly expressed in the liver of peak-laying hens than in that of juvenile chickens and were clearly less expressed in the kidney of peak-laying hens than in that of juvenile chickens, but no dramatic difference in their levels was found in the duodenum between the two stages. In birds, fat synthesis is extremely active in the liver at the stage of egg-laying (Macwhirter, 1999). The spatiotemporal expression of *NADB-LER*s in chicken liver showed that their expression was significantly up-regulated in the peak-laying stages relative to unmatured hens. It was suggested that *NADB-LER1, -2, -3, -4*, and *-5* may be strongly related to chicken hepatic lipid metabolism and play different roles in different tissues.

Estrogen, as a class of steroid sex hormone, is responsible for the development and regulation of the female reproductive system (Mohamadi et al., 2018). Generically, lipid metabolism is tightly regulated by estrogen in chicken with the onset of an egg-laying cycle (Hermier et al., 1996; Ivessa et al., 2013). Studies demonstrated that in estrogen-stimulated groups, the expression levels of *ApoVLDL II* and *ApoB* genes presented a dose-dependent increase (Miller and Lane, 1984; Hermier et al., 1989). Consistent with previous reports, our *in vitro* assay results indicated that 17β-estradiol significantly promoted the expression of *ApoVLDL II* and *ApoB* mRNA in chicken embryonic primary hepatocytes, suggesting that 17β-estradiol played a role in the hepatocytes. Meanwhile, *NADB-LER1*, *-2*, *-3*, *-4*, and *-5* were significantly up-regulated in 50 and 100 nM 17β-estradiol-treated groups. This result demonstrated that the expression of five novel genes could be controlled by 17β-estradiol in hepatocytes. Estrogen-inducible transcription regulation can be mediated via estrogen receptors (ERα and ERβ) or via intracellular G protein-coupled receptor 30 (GPR30) (Fietz et al., 2014), but the exact participation of the individual receptor is not clear. Our *in vitro* studies demonstrated that in chicken embryo hepatocytes, as a positive control, estrogen stimulates the expression of *ApoVLDL II* mRNAs predominantly through ER-α in the chicken liver, which were consist with previous report (Li et al., 2014). 17β-estradiol could significantly increase the expression of *NADB-LER1-5*, while the combined treatment of highly selective ERα antagonist MPP with 17β-estradiol almost fully blocked the effect.

In comparison with the MPP and 17β-estradiol-treated group, the combinations of ERα and ERβ antagonists ICI182,780 and tamoxifen with 17β-estradiol treatments failed to change the expression of *NADB-LER*s. It has been widely proven that tamoxifen, as a synthetic estrogen antagonist, represses target genes via binding to ERs at the transcriptional level (Lerner and Jordan, 1990) and that ICI 182,780 is a high-affinity ER antagonist (Turner et al., 2000). From these results, we concluded that estrogen activated the expression of *NADB-LER*s in chicken embryo hepatocytes mediated by ERα. The ER-mediated regulation of gene expression involves the direct binding of dimeric ER to DNA sequences known as estrogen response elements (EREs) (Klinge, 2001), or indirectly associate with promoters through protein-protein interactions with other DNA-binding transcription factors (Safe, 2001; Marino et al., 2006). To further sight into how estrogen regulated the expression of NADB-LER1-5 genes through ERα, the ERα specific EREs within the promoter region, 2.0 kb upstream the transcription start sites were predicated by using online software MEME Suite 5.1.0. The results showed that two putative ERα binding EREs occurred in the promoter regions of the *NADB-LER1* and *NADB-LER 3* genes, one occurred in *NADB-LER4*, and no one was found in *NADB-LER2* and *NADB-LER5*. Therefore, it suggested that the ERα dimer might activate the transcription of *NADB-LER1*, *-3* and *-4* genes by directly binding to EREs within their promoter, but activate the transcription of *NADB-LER2* and -*5* genes by binding to other transcriptional activators which indirectly regulated the genes’ transcription. However, we could not rule out the possibility that there were some ERα specific EREs existed in the promoter regions of *NADB-LER2* and -*5* genes further upstream.

During the chicken egg-laying stage, the increased production of VLDLs is accompanied by the upregulation of lipid synthesis and protein components, including *ApoVLDL II* and *ApoB* (Chan et al., 1976; Capony and Williams, 1980). The formation of hepatic VLDLs in avian is tightly regulated by estrogen, and therefore genes involved in the processes would be steered by estrogen. Previous reports demonstrated that estrogen administration to immature pullets (Heald and McLachlan, 1964) and roosters (Greengard et al., 1964) could lead to plasma changes similar to those occurring in the sexually mature female. In our study, the concentrations of TG, TC, and VLDL in the blood of 10 week pullets stimulated by 17β-estradiol hormone were dramatically increased, which is in accordance with previous references. In addition, the hormone caused massive synthesis of *ApoVLDL II* and *ApoB* in the liver tissue of chicken (Kirchgessner et al., 1987; Evans et al., 1988). Moreover, the *NADB-LER1-5* genes were significantly up-regulated by 17β-estradiol in the liver tissue of chicken, which further proved that *NADB-LER1-5* genes are likely involved in hepatic lipid metabolism under induction by 17β-estradiol in chicken.

Meanwhile, *NADB-LER1-5* genes were significantly inhibited in the kidney tissues of pullets under stimulation by 17β-estradiol. It is likely that chicken kidney could produce apoB-lipoprotein particles, but whether they are typical TG-rich particles or secreted into plasma is unknown (Kirchgessner et al., 1987). It was reported that bird kidney secretes generic VLDLs in some proximal tubule cells (Walzem et al., 1999). While the true biological role of *NADB-LER1-5* in chicken kidney is unclear, further studies will be needed to prove the biological function of the *NADB-LER* genes in chicken kidney.

## Conclusion

A novel gene family consisted of five novel genes *NADB-LER1-5* was identified in chicken genome. All of the genes in the family contain a common NADP-binding site that belongs to the NADB-Rossmann superfamily. The *NADB-LER* genes were mainly expressed in liver, kidney and duodenum and were significantly up-regulated in liver of hens in the peak-laying stage at the age of 30 and 35 weeks. The expression of the *NADB-LER* genes was predominantly controlled by17β-estradiol via ERα.

Taken together, our study suggested that the gene family may be a novel regulation factor involved in chicken hepatic lipid metabolism.

## Data Availability Statement

All datasets generated for this study are included in the article/Supplementary Material.

## Ethics Statement

This study was carried out in accordance with the recommendations of Animal Care and Use Guideline of Institutional Animal Care and Use Committee of Henan Agricultural University. The protocol was approved by the Institutional Animal Care and Use Committee of Henan Agricultural University (Permit Number: 11-0085).

## Author Contributions

HL performed the data analysis and drafted the manuscript. YL carried out the experiments. LY, DZ, and ZiL participated in the experiment. YW, RH, GL, and ZhL helped to analyze the data. YT and XK participated in the design of the experiments and the discussion. XL conceived the study, participated in the experimental design and helped to draft the manuscript. All authors read and approved the final manuscript.

## Conflict of Interest

The authors declare that the research was conducted in the absence of any commercial or financial relationships that could be construed as a potential conflict of interest.

## Footnotes

1 http://www.ncbi.nlm.nih.gov/BLAST

2 https://www.genscript.com/psort.html

3 http://www.cbs.dtu.dk/services/SignalP/

4 http://www.cbs.dtu.dk/services/TMHMM-2.0

5 http://www.cbs.dtu.dk/services/ProtFun

6 http://meme-suite.org/tools/meme

## Supplementary Material

FIGURE S1 Analysis of the conserved domains in the amino acid sequences of NADB-LER1-5. **(A)** NADB-LER1; **(B)** NADB-LER2; **(C)** NADB-LER3; **(D)** NADB-LER4; **(E)** NADB-LER5.

TABLE S1 Species and sequences selected for constructing the phylogenetic tree.
