# Supplementary figures and images for: Identification of a Novel Lipid Metabolism-Associated Hepatic Gene Family Induced by Estrogen via ERα in Chicken (Gallus gallus)
Source: Front Genet. 2020 Mar 31;11:271. doi: 10.3389/fgene.2020.00271 (PMC7136477; doi:10.3389/fgene.2020.00271)

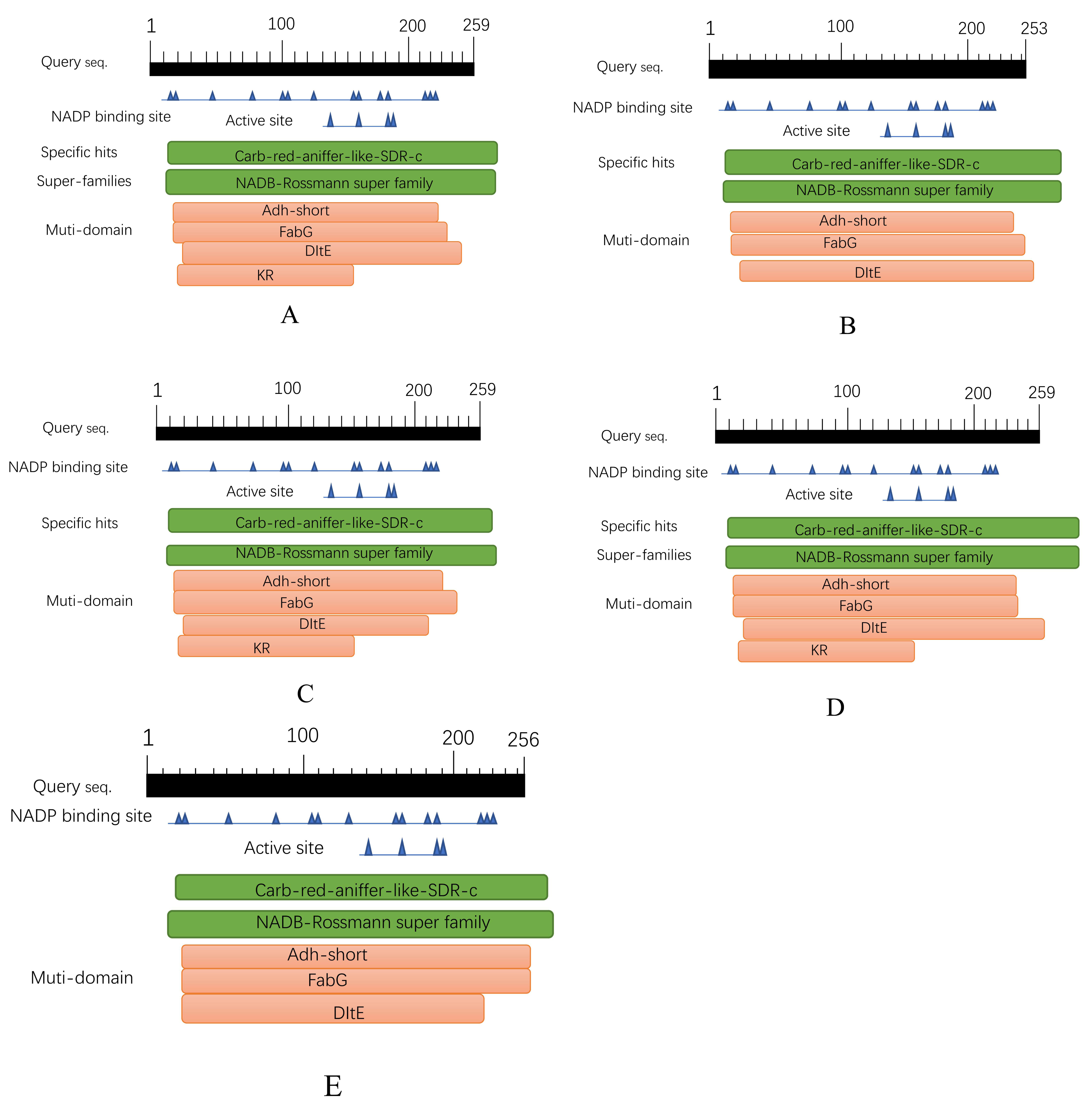

Supplement: FIGURE S1 — Analysis of the conserved domains in the amino acid sequences of NADB-LER1-5. (A) NADB-LER1; (B) NADB-LER2; (C) NADB-LER3; (D) NADB-LER4; (E) NADB-LER5. [file Image_1.TIF]
