# Supplementary material for: Identification of a Novel Lipid Metabolism-Associated Hepatic Gene Family Induced by Estrogen via ERα in Chicken (Gallus gallus)
Source: Front Genet. 2020 Mar 31;11:271. doi: 10.3389/fgene.2020.00271 (PMC7136477; doi:10.3389/fgene.2020.00271)
Supplement: TABLE S1 — Species and sequences selected for constructing the phylogenetic tree. [file Table_1.DOCX]

Table.S1 Species and sequences selected for constructing the phylogenetic tree

| Species name | Gene ID | | | | |
| --- | --- | --- | --- | --- | --- |
|  | *NADB-LER1* | *NADB-LER2* | *NADB-LER3* | *NADB-LER4* | *NADB-LER5* |
| Gallus gallus | ENSGALG00000001824 | ENSGALG00000023936 | ENSGALG00000021451 | ENSGALG00000021450 | ENSGALG00000001791 |
| Turkey | ENSMGAT00000002344 | ENSMGAT00000003120 | ENSMGAT00000002528 | ENSMGAT00000002310 | ENSMGAT00000002520 |
| Duck | ENSMGAT00000002587 | ENSAPLT00000007242 | ENSMGAT00000002540 | ENSAPLT00000008674 | ENSAPLT00000007260 |
| Spotted gar |  | ENSLOCT00000002903 |  |  |  |
| Cave fish |  | ENSAMXT00000004621 |  |  |  |
| Flycatcher | ENSFALT00000007453 | ENSFALT00000009745 | ENSFALT00000007535 | ENSFALT00000007458 | ENSFALT00000007470 |
| Chinese softshell turtle | \| ENSPSIT00000004835 \| \| --- \| |  |  |  |  |
| Zebra finch |  |  | ENSTGUT00000005775 |  | ENSTGUT00000005777 |
